# Notes on the use and interpretation of radiostereometric analysis

**DOI:** 10.1080/17453670902807474

**Published:** 2009-02-01

**Authors:** Brian Derbyshire, Robin J Prescott, Martyn L Porter

**Affiliations:** ^1^Centre for Hip Surgery, Wrightington HospitalAppley BridgeUK; ^2^Medical Statistics Unit,Public Health Sciences, University of Edinburgh Medical SchoolEdinburghUK

## Abstract

**ABSTRACT** With increasing numbers of research groups carrying out radiostereometric analysis (RSA), it is important to reach a consensus on how the main aspects of the technique should be carried out and how the results should be presented in an appropriate and consistent way.

In this collection of guidelines, we identify a number of methodological and reporting issues including: measurement error and precision, migration and migration direction data, and the use of RSA as a screening technique. Alternatives are proposed, and a statistical analysis is presented, from which a sample size of 50 is recommended for screening of newly introduced prostheses.

## Introduction

Radiostereometric analysis (RSA) is being increasingly used for assessment of the 3-dimensional migration patterns of orthopedic implants, particularly total joint prostheses. The high precision of the technique has enabled just a small sample of patients (< 30) to be followed up over a 2- or 3-year period. The working hypothesis is that by the end of this period, a prosthesis showing continuous excessive migration would be likely to fail in the medium to long term (Kärrholm et al. 1994, Ryd et al. 1995). This extrapolation from the short term to the long term has enabled RSA to be considered for short-term screening of new joint prosthesis designs before they are commercially deployed.

As Valstar et al. (2005) have pointed out, there is a need for standardization of the RSA procedure among the increasing number of research groups. At present, each RSA research group has its own way of determining and presenting results. The results may be from single-prosthesis studies or randomized studies, sample sizes may differ between groups, and results may be presented in different ways (e.g. mean, median, mean absolute, migration rate) and with different “failure” thresholds (e.g. mean ± 2 SD).

In this paper we examine, question, and offer alternatives to some of the current practices of RSA and its presentation. Topics include measurement error and determination of precision, appropriate methods of presenting summaries of migration and direction of migration, and the use of RSA as a screening technique. We present a statistical analysis to determine an appropriate sample size for screening of prostheses.

### Measurement error

The measurement error of an instrument can have 2 sources: systematic and random. Systematic error (bias) is instrument-, technique-, or observer-dependent and it can be constant or vary with the magnitude of the measurement. The RSA technique involves a variety of “instruments” (radiographs, calibration table, digital scanner, software algorithms), and systematic errors may be present in all of these. Modern RSA software has effectively eliminated the subjective, observer component by employing semi-automated determination of marker centers. Since an RSA measurement is the result of a difference between the reference (e.g. postoperative) and current examination recordings, most of this systematic error should disappear in the subtraction process, and so this error component should be zero or very small. Its magnitude can only be determined from phantom measurements in vitro, where RSA migration measurements can be compared with “exact” settings. Studies have shown it to be just a few hundredths of a millimeter or degree in a plane parallel to the radiographic plane, and slightly more in the out-of-plane direction (Bragdon et al. 2002, Von Schewelov et al. 2004).

Other types of bias will also affect the results. These could be caused by, for example, patient factors (e.g. weight, activity level) or variations in the surgical technique (including the number and scatter of bone markers), postoperative rehabilitation protocol, or time of the reference (index) radiographic examination. For instance, with a “constrained” (Derbyshire and Porter 2007) cemented hip stem or an uncemented hip stem, which would be effectively “loose” immediately after surgery, a large amount of migration would occur in the first few postoperative days as the stem settled in, and this would be affected by the rehabilitation protocol. If the time of the reference radiographic examination was just a few days late, much of the early migration would be “lost” from the recordings. Strictly controlled processes and protocols are therefore required to reduce this type of bias, but it cannot be eliminated. This is perhaps the main reason why comparing the direct magnitudes of migration could be misleading, especially when comparing the results from different studies (Derbyshire and Porter 2006). It might therefore be better to assess the rates of the main components of migration after, say, 2 or 3 years (perhaps by using a paired t-test to determine whether a significant difference in migration had occurred during the previous 12 months) because the migration of “good” prostheses would be expected to have levelled off by that time. Any excessive departures from the mean rate (> 2 SD from the mean, say) would then identify those prostheses that were not “behaving” as expected. If the mean rate at that time was considered to be higher than expected, the study could be extended by (say) another year—and if it was still high then, the prosthesis would be considered to be suspicious. It is relevant to point out at this juncture that, for constrained (tapered, polished, collarless) hip stems, there is no agreed threshold level of migration above which failure might be predicted. For these designs, measurement of the migration rate after 2 or 3 years is probably our best method of assessment.

One way of overcoming these various types of bias is to carry out a randomized clinical trial of two prosthesis designs, in which the migration magnitudes are compared. In such a study, randomization ensures that other variables are distributed between the two groups without bias, but it is important to realize that this is not the same as ensuring that they will be identically distributed. Furthermore, the sample size would need to be appreciably larger than that required for a study of just one group. Even if the randomized trial determines statistically significantly more migration in the test group, we still have the problem of the clinical relevance of that increased migration. However, if we are simply interested in screening (see below), it might suffice to assess one particular design, recording the proportion of prostheses showing relatively high migration rates (> mean + 2 SD) after 2 years.

The random error component affects the precision of the measurement. It can be estimated using an in vitro phantom (Kärrholm et al. 1997, Ryd et al. 2000, Onsten et al. 2001, Börlin et al. 2002, Bragdon et al. 2002, Makinen et al. 2004, Von Schewelov et al. 2004) but, since random effects are brought into play for each particular radiographic set-up and each patient, it is best assessed from patient radiographs using the “double-examination” technique. This involves repeating the RSA examination on a sample (or all) of the patient cohort. For a double examination to fully simulate the clinical measurement errors, the two radiographic tubes, the calibration table (if not fixed in position), and the examination table should be moved and then re-set during the interval between the two examinations (while the patient has left the room). Although researchers generally report the results of double examinations, it is not always clear that they have followed the latter procedure—which can make a substantial difference to the measurements (Makinen et al. 2004).

The statistical method for dealing with the repeated measurements of double examinations has been described (Ranstam et al. 2000). The repeatability is determined in terms of the standard deviation of the differences between a series of double measurements. If the bias is assumed to be zero, the population mean of the random error will be zero. As Ranstam et al. (2000) have pointed out, in such a case the mean is not estimated and the denominator in the SD formula should therefore be n, and not n – 1. The numerator in the expression for the SD will be the sum of squares rather than the sum of squares about the sample mean. To obtain tolerance limits that would contain 95% of the possible errors, they multiplied the above SD by 1.96, which is in accordance with the ISO standard. This assumes a normal distribution and a large sample. If only a small number of patients (say < 30) have undergone double examinations, it is important that a Student’s t distribution with n degrees of freedom be used to calculate the 95% tolerance limits, and the SD multiplied by a “t” statistic instead. Even with larger sample sizes, this approach is still preferable, though its impact is smaller. The resulting value gives the possible error between any 2 measurements in a series. The accuracy variance is half the repeatability variance (Ranstam et al. 2000), and so the accuracy would therefore be given by t × SD / √2. This refers to the accuracy of an individual measurement. In the context of double measurements, this is a notional concept rather than a useful measure.

The assumption implicit in the above approach to the double measurements is that the repeatability is independent of the amount of migration that has occurred relative to the reference recording. This assumption will often be reasonable, but it can be checked graphically by plotting the differences against the absolute value of the mean. If the assumption holds, there should be no association.

### Mean and resultant migration

Calculation of a resultant requires that all vectors act on the same object: in concert (when the vectors are acting at the same instance) or serially (when each vector acts at a different instance). The resultant of several vectors gives the final outcome of those vectors, but it cannot be calculated simply by summing the displacement magnitudes; the directions must also be taken into account using trigonometric calculations.

A displacement vector acting on a prosthesis in one patient would necessarily be independent from a vector acting on a prosthesis in another patient. Since the 2 vectors would not be acting on the same object, it would not be valid to calculate their resultant. To summarize the overall migration of several prostheses, a mean value must be calculated in some way, but this is not straightforward.

**Figure 1.**
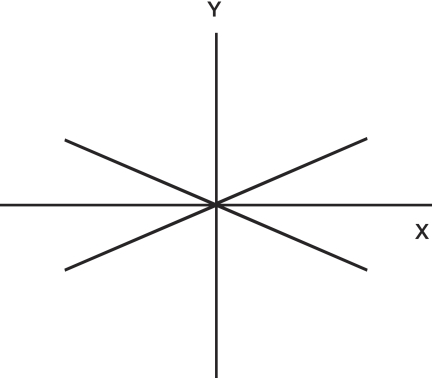
Four displacement vectors are shown in the x–y plane. Each is symmetrically distributed about the x-axis and the y-axis. The mean of the vector components in the x- or y-directions would be reported as zero even though each individual vector is much greater than zero.

For statistical purposes, it is valid to gather all of the x-components of translation (say) into a collection of positive and negative scalar quantities and to calculate a mean. The summary statistic may be misleading, however. For the 4 vectors shown in Figure 1, the sum—and therefore the mean—of the scalar components in the x-direction is zero. However, none of the vectors or their components is even close to zero. The results might therefore be reported something like this: “overall, there was no migration in the mediolateral direction”. The zero mean therefore tells us nothing about the mean magnitude of the displacement vectors (or their components) acting on all of the prostheses. The standard deviation would give an indication of the dispersion of the scalars, but this is not as informative as a mean magnitude of the vectors.

Two ways of overcoming this problem are: (a) to find the mean of the scalar magnitudes of the vectors—not vector components that have been resolved in the direction of the x-, y-, and z-axes as mentioned above (see Direction of Migration below); (b) to find the mean absolute scalar magnitude of the vector components along each axis (x and y in the above example). A recent paper proposing guidelines for the standardization of RSA has recommended that absolute values should not be used (Valstar et al. 2005). The main reason for this recommendation was that absolute migrations are not normally distributed, and it was stated that confidence intervals for the mean could not therefore be calculated. In fact, the parametric methods for calculation of confidence intervals for the mean are remarkably robust unless the distribution is extremely skewed and the sample size inadequate (Kirkwood and Sterne 2003). We carried out a bootstrapping technique (Efron and Tibshirani 1993) on absolute RSA migration data of 3 translation and 3 rotation components of 19 cemented hip stems measured at the 2-year examination, and then compared the results with those computed using a standard statistical technique (t-distribution). We confirmed that the mean and its confidence interval could be found to a high degree of accuracy using the standard technique. Evaluation of the mean of absolute values and its confidence limits gives a good indication of the general size of the vector components, which is not diminished (due to cancelling) by sign/direction considerations.

### Direction of migration

**Figure 2.**
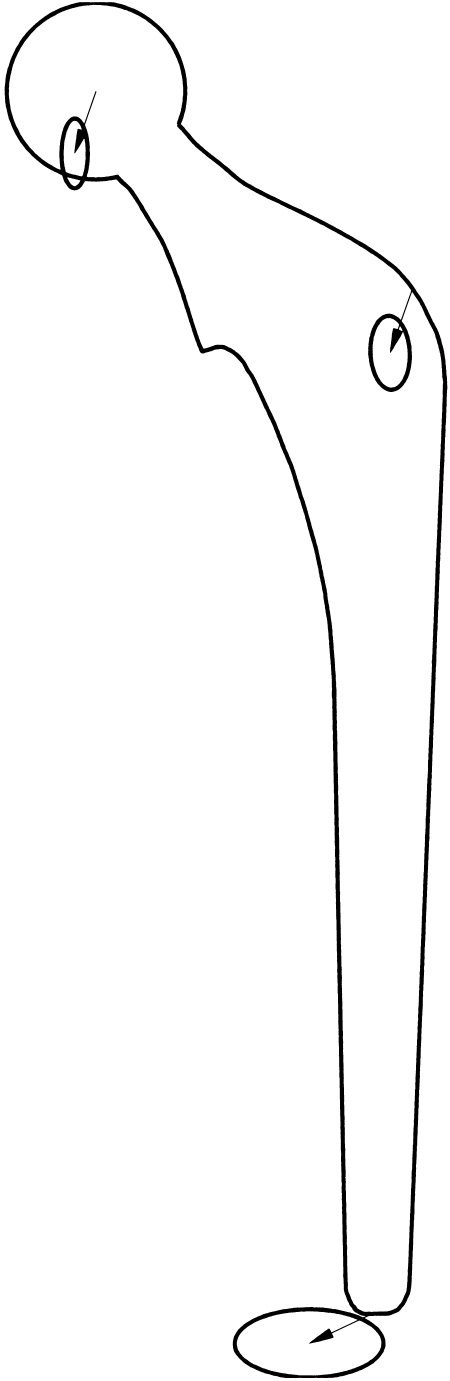
Diagram showing how the direction of the mean migration has been reported by others. The length of the line at each landmark signifies the mean migration and its angle gives the direction of the mean (see text). The magnitude of the ellipse is related to the standard error.

**Figure 3.**
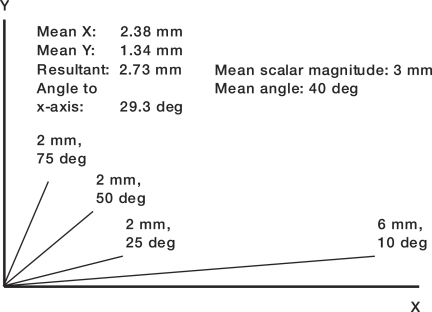
Diagram showing why the resultant of several vectors cannot be used to determine the mean direction of the vectors. The mean direction calculated using the resultant (i.e. 29 degrees) is dominated by the largest (6-mm) displacement vector. The true mean direction angle is 40 degrees.

**Figure 4.**
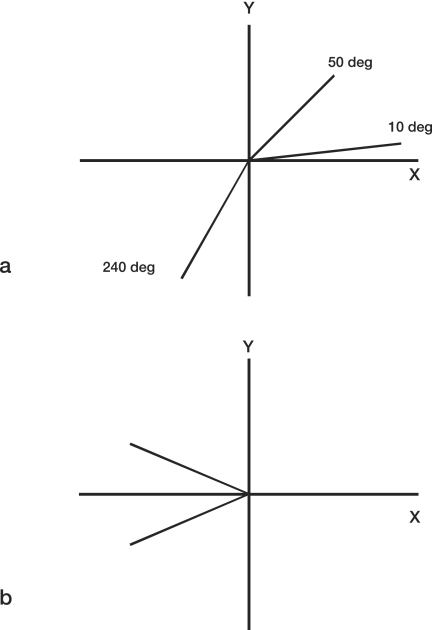
(a) The mean direction cannot be determined as the mean of the vector angles from the positive x-axis; here, this would be 100 degrees, but the mean direction should be in the bottom right quadrant. (b) If the angle is measured in positive and negative (clockwise) directions with respect to the positive x-axis, the mean can still not be determined; here, it would be zero degrees, which is incorrect.

Some papers (Kiss et al. 1995, 1996, Alfaro-Adrian et al. 1999, 2001, Catani et al. 2005) have included diagrams of a hip stem as viewed from different directions, and a resultant translation vector has been drawn at each of the measurement landmarks. The direction of the resultant has been determined from the mean (or median: Catani et al. 2005) displacement vector component in each of 2 axis directions. Thus, in the x–y plane, when mean values were used, the angle to the x-axis was found by tan^-1^(mean y / mean x). In addition, the standard errors of the mean x and mean y (or the upper and lower quartiles) have been represented as the major and minor axes of an ellipse drawn at the end of each resultant vector (Figure 2). This method might not provide a good estimate of a representative direction of translation. It would place heavier weight on those prostheses with the largest scalar changes. For example, in Figure 3, the 6-mm vector dominates the direction of the resultant, reducing it to less than 30° to the x-axis. The true mean direction is 40°, i.e. (25 + 50 + 75 + 10) / 4, and the mean scalar magnitude is 3 mm, i.e. (2 + 2 + 2 + 6) / 4. The whole concept of calculating the mean direction has to be used with caution, however. It cannot be used in this simple way when the vectors are spread over a 360° range. The following examples show why this is so. In Figure 4a, 3 vectors are at 10°, 50°, and 240° to the positive x-axis. The mean angle would therefore be 300° / 3, i.e. 100°. This is incorrect; the mean should be in the bottom right quadrant. If the angle of the vector at 240° is calculated in the negative, clockwise direction, i.e. –120°, the mean angle would be –60° / 3, i.e. –20°, which appears to be correct. However, using positive and negative directions like this does not work: the mean angle of the 2 vectors in Figure 4b would be zero, which is clearly incorrect.

**Figure 5.**
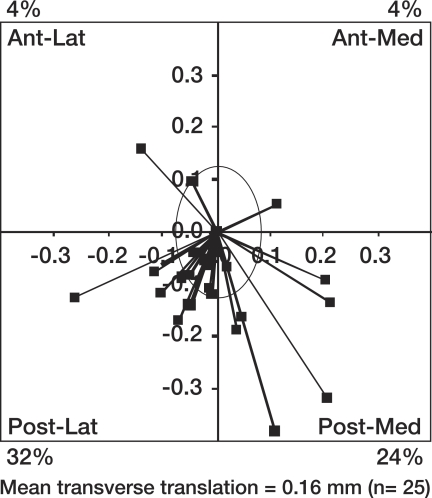
Vector plane chart (created using RSA DataViewer: www.orthomech.co.uk) showing all the vectors in the transverse plane at the 6-month stage of a cemented hip stem study. The ellipse shows the variation of the resultant of the repeatability (precision) error in the transverse (x–z) plane. The intersection of the ellipse with the x-axis (mediolateral) and z-axis (anteroposterior) is the precision (determined from double examinations) in the x- and z-directions, respectively. A summary of the vector directions is given by the percentage of vectors (beyond the repeatability error, i.e. outside the ellipse) in each direction quadrant. In this case, 36% of the vectors were within the ellipse and they were not therefore considered in the calculations of the proportions in each quadrant. The mean scalar value of all the vectors in that plane is shown at the bottom of the chart.

A simple descriptive approach is to plot the individual translations in a specified plane. Figure 5 shows stem centroid translation vectors at 6 months in the transverse (x–z) plane for 25 cemented femoral stems. Each translation vector is represented by a single point, the line to each point emphasizing the magnitude and direction of the vector with respect to the immediate postoperative position. The point is determined from the 2 orthogonal translation components in the plane of interest (the x–z plane in Figure 5), with signs according to the medial/lateral, anterior/posterior, proximal/distal directions (medial, anterior, and proximal positive). The magnitude of each vector is the length of the line (i.e. the square root of X^2^ + Z^2^ in this case). Its direction can be reported in terms of the anticlockwise angle to the horizontal, right axis. The directions of the vectors have been summarized as a proportion of vectors in each quadrant, expressed as a percentage. The mean scalar magnitude of all the vectors (line lengths) in that plane is presented at the base of the graph. In the text, a confidence interval for this mean could be included. Where necessary, this sort of chart could be presented for the 2 other planes. A plane chart at a particular examination could be presented as a supplement to standard migration-time charts.

Some of the translation vectors in a plane might be within measurement error. The chart could therefore be improved by including “error bounds” enclosing an area about the zero datum, inside which the “unmeasurable” vectors would be contained. The +/- error threshold would then take the form of an ellipse derived from the resultant of the double examination measurements in that particular plane (see above). The proportion (%) could then relate to just those vectors outside the bounds of the ellipse (Figure 5). (Note 1: if the double examination errors in the x- and z-directions (for instance) are found to be Ex and Ez, respectively, the magnitude of an error vector in the x–z plane at an angle θ to the x-axis would vary between E_x_ and E_z_. For E_x_ = E_z_, it would remain the same for all values of θ, i.e. the vector would describe a circle. If E_x_ ≠ E_z_, the locus of the vector over the range of θ would describe an ellipse having extreme “radii” E_x_ and E_z_. Note 2: the calculation of the mean translation should not exclude those vectors within the ellipse, as this would artificially and unfairly inflate the mean).

For the case of rotations, these can only be realistically presented with respect to the 3 axes of rotation. If the mean absolute rotation about a particular axis was presented, it would be informative also to present the direction (and number of prostheses) in which the majority had rotated.

### RSA as a screening device

In a variety of orthopedic studies, RSA has been employed simply as an accurate 3-dimensional measuring device. However, its increase in popularity over the past 10 years has undoubtedly been due to its potential as a screening device for prosthetic joints. An association between early excessive migration and medium-to-long-term loosening of prosthetic joints was suggested by studies carried out in the 1990s (Freeman and Plante-Bordeneuve 1994, Kärrholm et al. 1994, Ryd et al. 1995, Kobayashi et al. 1997). It was hypothesized that the medium- to long-term outcome of a joint prosthesis could be predicted by measuring early migration. These studies elevated the clinical relevance of migration measurements and introduced the possibility of screening newly introduced prostheses. Freeman et al. (1994) wrote: “We believe that any new femoral prosthesis should be monitored on introduction, by measuring its migration rate in a small number of patients over a period of 2 years… rapidly migrating components should be abandoned forthwith.” When RSA was subsequently employed, the increased measurement accuracy and precision increased the predictive power. Ryd et al. (1995) reported a predictive power of 85% for identifying “at risk” total knee prostheses 1–2 years after operation. Kärrholm et al. (1994) found that the probability of femoral stem revision was more than 50% if femoral head subsidence was greater than 1.2 mm at 2 years. The scene was set for RSA to be considered as a screening technique for new joint prostheses.

Two important points must be considered in relation to screening. The first relates to sample size. The second relates to whether RSA alone can identify prosthesis designs that are likely to have a poor long-term outcome.

**Figure 6.**
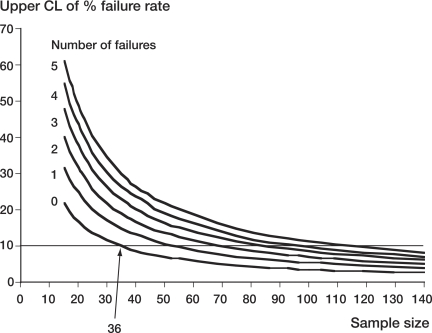
Variation of the upper confidence limit of the population percentage failure rate with sample size for different numbers of failures in the sample. The horizontal line indicates a 10% threshold of acceptability for the population failure rate. Even if there were no predicted failures in the RSA sample, a minimum sample size of 36 would be required for the upper confidence limit to fall within the 10% threshold. If there was just 1 predicted failure, the sample size would have to be at least 54.

As mentioned in the Introduction, due to the high precision of RSA, excessively migrating prostheses can be discriminated, and failure predicted, using only a small sample size: about 15–25 patients (Valstar et al. 2005). It should be emphasized, however, that the prediction of failure is for the excessively migrating prostheses in that sample only. With such a small sample, an RSA study would not enable accurate predictions about the general outcome of a prosthesis design, i.e. its population failure rate. This is because the confidence interval for the estimate of the failure rate would be too wide. Only grossly unsuitable prostheses would be reliably identified from such studies. Figure 6 shows how the upper confidence limit of the estimated failure rate varies with the number of patients in the study sample for different numbers of predicted failures. If, as mentioned above, the sample size was 15, then a failure rate of 1 in 15 (7%) for the sample would have a 95% confidence interval of 0.2–32% for the inferred population failure rate (Clopper-Pearson exact proportion, 95% CI, using StatsDirect software; StatsDirect Ltd., Altrincham, UK). The estimate therefore has no practical value.

In the design and interpretation of a screening study, there are two considerations: a prosthesis design should be safe; and “safe” prostheses should not be predicted to fail. A benchmark revision rate of 10% at 10 years is generally considered to be the threshold of acceptability for primary total hip replacement (see, for instance, NICE 2000). In the foregoing analysis, a 10% revision rate following aseptic loosening will be assumed.

It can be seen from Figure 6 that in order to safely estimate (i.e. with 95% confidence) that the predicted population failure rate would be 10% or less, a minimum sample size of 36 would be required, even if there were no predicted failures in the sample. Figure 6 also shows that the occurrence of one or more predicted failures in the sample would require the sample size to increase even further if the 10% benchmark criterion were to be met. Thus, if 1, 2, 3, 4, or 5 failures were suspected in an RSA study, the associated sample sizes would have to be 54, 70, 85, 100, or 114, respectively, in order for the prosthesis design to meet the benchmark.

The above failure requirement would impinge upon the second consideration: measurements from the RSA sample should not suggest that “safe” prostheses would be likely to fail. In this context, a safe prosthesis could be defined as one of the best performers in arthroplasty register survival tables. This amalgamated experience of survivorship data from arthroplasty registers is probably the closest we will ever get to designating a prosthesis as “safe”—in the sense of a low probability of failure over a long period, i.e. not “unsafe”. Thus, if a population failure rate at 10 years of, say, 3% (97% survival rate) was chosen as the threshold of safety, cemented femoral components such as the Exeter and Lubinus SP II would fall within this criterion (Danish and Swedish Arthroplasty Registers, Annual Report 2005). However, even with a 3% population failure rate, there would be a 67% chance that 1 or more prostheses in a sample of 36 subjects would be predicted to fail (Table 1). Using the “standard” method of sample size calculation, a sample of 100 would be required to ensure (with 80% power) that prostheses similar to the top performers would not be rejected when the true population failure rate was 3% (Table 2). Thus, even with a modest safety threshold of 3%, the required sample size would be prohibitively large for most manufacturers. A compromise to the above analysis, which might provide a better safeguard without financially stretching the orthopedic implant industries, would be to demand observed failure rates below the benchmark of 10% (≥ 90% survival rate), with upper 95% confidence limits for failure rates less than 15%. The “safe” prosthesis design, with a population failure rate of 3% (or less) would then require only 46 prostheses to be evaluated while ensuring an 80% probability that an RSA study would not suggest that it might fail.

**Table 1.** This table shows that even a well-performing femoral stem design (as determined from arthroplasty registers) could have a high probability of being designated as a possible future failure by an RSA study. Left column: here, a well-performing femoral stem design is considered to have a population failure rate of up to 5% (aseptic survival rate: 95%). The probabilities of RSA revealing possible failures (excessively migrating stems) in samples of 36 and 54 of such stem designs are shown in columns 2 and 3.

Failure rate	P (≥ 1 in 36)	P (≥ 2 in 54)
5%	84%	76%
4%	77%	64%
3%	67%	48%
2%	52%	29%
1%	30%	10%

**Table 2.** In order to have an 80% chance that RSA would not reveal possible failures (excessively migrating stems) in a test sample of well-performing femoral stems (as denoted by the low failure rates in the left column), the RSA sample size would have to be as shown. Columns 2 and 3 show the minimum sample size required when the upper 95% confidence limit of the failure rate is 10% or less (the generally accepted failure rate benchmark for hip prostheses at 10 years). Columns 4 and 5 show the sample size when this confidence limit is raised to 15%. The effect of using a 1-tailed or 2-tailed test for the sample size calculation is also shown

	Required sample size (80% power)			
	Upper 95% CL ≤ 10%		Upper 95% CL ≤15%	
Failure rate	2-tailed	1-tailed	2-tailed	1-tailed
5%	231	179	75	59
4%	154	116	56	50
3%	100	76	46	40
2%	70	61	35	30
1%	54	46	35	19

The question of whether RSA can be used to screen new prosthesis designs also depends on whether a particular aspect of the design can be identified as a cause of excessive migration. This might not be straightforward. One approach is to eliminate other known causes of, or contributors to, excessive migration. To do this, as many patient and surgical factors as possible must be measured or assessed and then compared to accepted standards. High “migrators”, judged to have been caused by any of these factors, would not be considered when predicting the population failure rate from the RSA sample failure rate.

Although the analysis above shows that a sample size of 46 would be required for a screening study, it would be advisable to inflate the sample size above this minimum in anticipation of RSA failures that were unrelated to prosthesis design. We therefore suggest that a minimum sample size of 50 would be appropriate. There is an urgent need for the manufacturers, the regulators, and the research community to come together to agree on the statistical objectives of trials using RSA methodology and to provide clear guidelines for their implementation.
